# Incident cervical infections with high- and low-risk human papillomavirus (HPV) infections among mothers in the prospective Finnish Family HPV Study

**DOI:** 10.1186/1471-2334-11-179

**Published:** 2011-06-22

**Authors:** Karolina Louvanto, Marjut A Rintala, Kari J Syrjänen, Seija E Grénman, Stina M Syrjänen

**Affiliations:** 1 Medicity Research Laboratory and Department of Oral Pathology, Institute of Dentistry, Faculty of Medicine, University of Turku, Lemminkäisenkatu 2, 20520 Turku, Finland; 2 Department of Obstetrics and Gynecology, Turku University Hospital, University of Turku, Kiinanmyllynkatu 4-8, 20521 Turku, Finland; 3 Department of Oncology and Radiotherapy, Turku University Hospital, Hämeentie 11, 20500 Turku, Finland

## Abstract

**Background:**

The knowledge on type specificity and factors that increase or decrease the risk of incident HPV-infections is important to better understand the dynamics of HPV-infections.

**Methods:**

A series of 329 pregnant women were enrolled in Finnish Family HPV Study at 3^rd ^trimester of pregnancy and followed-up for 6 years, during which 203 baseline HPV-negative women acquired incident HPV infection. Incidence times and incidence rates (IR) were calculated for 24 low-and high-risk HPV-types detected by Multiplex-HPV-genotyping at each visit. Poison regression was used to estimate predictors of incident HPV infections of species 7 and 9 HPV-genotypes.

**Results:**

HPV16 was the most frequent (47.8%) incident genotype followed by multiple-type infections (25.1%), and single infection with HPV18, 70, 6 and 45. Actuarial mean times to incident event were longest for HPV31 (34.5 months) and HPV45 (32.8 months), while crude mean times were longest for HPV56 (42.4 months) and HPV16 (23.1 months). Actuarial IR was highest for HPV16 and multiple-type infections. Independent protective factors against incident infections were 1) > 2 life-time sexual partners (p = 0.014), 2) later initiation of oral contraceptives (age > 20 years) (p = 0.017) and 3) pregnancy at FU visit (p = 0.0001).

**Conclusions:**

Among newly delivered mothers, higher number of life-time sexual partners, initiation of OC use after age 20 and becoming pregnant during FU decreased the risk for incident species 7/9 HPV infections.

## Background

Human papillomavirus (HPV)-types that infect the female genital tract belong to the alpha-papillomavirus-genus which includes 15 species and 58 HPV-genotypes. According to their clinical behavior, 15 HPV-types are high-risk (HR)-types, 12 are low-risks (LR)-HPV-types, and three probable HR-types [1,2]. Worldwide, the eight most common HPV-types in cervical cancer are all included either into species7 (HPV18,45) or 9 (HPV16,31,33,35,52,58) [3]. The studies on incident HPV-infections have been evaluating HR- and LR-types collectively or HPV in general [4-7] or acquisition of incident HPV-infections have been assessed at genotype level [8-16].

Among young women, incident infections with HR-HPV-types especially with HPV16 [8-11,14-16] seem to be more common than LR-types [9,17]. The knowledge on type specificity and factors that increase or decrease the risk of incident HPV-infections is important to better understand the dynamics of HPV-infections and to take appropriate measures for their optimal prevention.

The aim of this study was to assess the frequency of type-specific incident HPV-infections in addition to actuarial and crude incidence-times and -rates of the most common LR- and HR-HPV- genotypes in newly delivered mothers, prospectively followed-up for 6 years in the Finnish Family HPV Study. Risk-factors of incident HPV-infection were analyzed with univariate and multivariate Poisson regression for panel data.

## Methods

### Subjects

The Finnish Family HPV Study is a prospective cohort study conducted at the Department of Obstetrics and Gynecology, Turku University Hospital and the Institute of Dentistry, Faculty of Medicine, University of Turku. The study was designed to evaluate the dynamics of HPV infections in mothers, fathers and their newborn infants. A total of 329 women, 131 men and 331 children were recruited to this study between 1998 and 2002. An extended 6 years follow-up was performed between 2006 and 2008, and invitation to this follow-up visit was send to all women and men in this cohort; a total of 171 women and 44 men was finally reached and examined.

**Figure 1:**
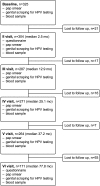
**Flow chart of the mothers included in the Finnish Family HPV study**.

Subjects in this cohort comprise mothers-to-be who were recruited at the minimum of 36 weeks of pregnancy [18] and followed-up for up to 6 years (Mean = 54.9 months, SD = 27.3, Median = 62.4; range: 1.6-94.5 months) after the delivery. The Research Ethics Committee of Turku University Central Hospital has approved the study protocol and its amendment (#2/1998 and #2/2006) and an informed consent have been gained from all the participants of this study. Altogether, 329 mothers were recruited (mean age 25.5 years, SD = 3.6 years), of whom 203 baseline HPV-negative women (mean age 25.48 years, SD = 3.1 years, range 18-38) who developed an incident event during the follow-up (FU) were included in this study. The women of this study are of Caucasian origin, have the same ethnic background and are representative of the Finnish population. The flowchart of the study setting is described in Figure 1. Some of the study participants were lost to follow-up, mainly due to difficulties in getting to study appointments or lack of child care. Structured questionnaire for recording demographic data and potential risk factors (i.e. sexual behavior, gynecological and obstetric history and risk factors for HPV infections) were recorded at the 2-month and 6-year FU-visits. During the 12-month and 36-month FU-visit, the data on the presence of HPV-induced clinical lesions, such as oral papilloma, skin and genital warts were recorded. Selected data from these records were used for risk assessment in the present study, as separately indicated.

### Samples

The scrapings for HPV-testing were taken at the baseline and at 2-,12-,24-,36-month and 6-year FU-visits. Sampling was done with a cytobrush from the uterine cervix (Cytobrush, MedScand, Malmö, Sweden) using sampling media of 0.05 M phosphate-buffered saline with 100 μg gentamycin. The samples were immediately frozen at -20°C and stored at -70°C. Scrapings from the oral mucosa were also collected at each visit [19], but only the baseline DNA data were used in the present study.

### Pap smears

A routine pap smear was taken from all women at baseline, and 2-,12-,24- and 36-month, by using conventional three-sample technique with wooden spatula and cytobrush (Medscand, Malmö, Sweden) as described earlier [18].

### HPV-genotyping

The HPV genotyping was done by multiplex-HPV-genotyping kit (Multimetrix, Progen Biotechnik GmbH, Heidelberg, Germany) as outlined in our recent study [20]. The kit identifies the following 24 LR- and HR-HPV-genotypes: LR-HPV: 6,11,42,43,44,70; HR-HPV: 16,18,26,31,33,35,39,45,51,52,53,56,58,59,66,68,73,82 [21]. With Luminex LX-100-analyzer (Bio-Plex 200 System, Bio-Rad Laboratories, Hercules, USA) medium fluorescence intensity (MFI) of at least 100 beads was computed for each bead set in the sample. The cut-off value for each run and HPV-type was 1,5x background MFI (negative control)+5MFI.

HPV genotyping was done using the earlier PCR product which was now re-amplified with GP05+ and bio-GP06+-primers [22]. Originally DNA was extracted from scrapings with high salt method [23]. The PCR products were then hybridized with digoxigenin-labelled HR-HPV oligoprobe cocktail (HPV-types 16,18,31,33,35,39,45,51,52,54, 56 and 58) to detect the presence of any high risk HPVs [24].

As HPV16 is the most prevalent genotype, we retested all HPV16-positive samples as described by Schmidt and co-workers [21] and discounted the possibility of false positive samples due to contamination.

### Serology

Blood samples were taken at baseline and at 2-,12-,24-and 36-month and 6 years of FU. Samples were analysed for the antibodies to the major capsid protein L1 of HPV types 6,11,16,18 and 45, and were analysed by multiplex HPV serology based on glutathione S-transferase fusion-protein capture on fluorescent beads. These results were interpreted using the technique and cut-off values as recently described [25]. The Selected data from these records were used for risk assessment in the present study (Table 1).

**Table 1:** Predictors of species 7 and 9-type specific incident^a ^HPV infections^b^

Covariates	Incident Species 7 and 9 HPV Infections					
	**Crude IRR**	**95% CI**	**P**	**@Adjusted IRR**	**95% CI**	**P**

Age (at study entry; continuous var)	*0.98*	*0.96-1.02*	*0.090*	0.99	0.98-1.01	0.678
Mother seroconverted to HR-HPV						
No	Ref					
Yes	**0.81**	**0.67-0.98**	**0.039**	0.84	0.69-1.03	0.106
Mother seropositive to HR-HPV at baseline						
Yes	Ref					
No	1.10	0.85-1.42	0.463			
Baseline oral HR-HPV DNA status						
HR-HPV-	Ref					
HR-HPV+	0.80	0.54-1.19	0.285			
Baseline PAP smear						
ASCUS+	Ref					
Negative (WNL)	1.76	0.76-4.10	0.184			
Marital Status at baseline						
Singe	Ref					
Married	1.19	0.77-1.81	0.419			
Living with partner	1.30	0.86-1.97	0.208			
Divorced	1.42	0.95-2.15	0.086			
Employment status						
Employed	Ref					
Student	0.82	0.61-1.10	0.192			
Unemployed	0.90	0.75-1.07	0.257			
Age at onset of sexual activity						
Below 13 years	Ref					
Above 13 years	**1.15**	**1.05-1.27**	**0.003**	1.05	0.93-1.17	0.394
No. of sexual partners until age of 20 yrs						
0-2 partners	Ref					
3-5 partners	0.89	0.76-1.05	0.201			
6-10 partners	1.05	0.92-1.19	0.410			
> 10 partners	0.73	0.46-1.18	0.206			
Life-time number of sexual partners						
1-2 partners	Ref					
3-5 partners	**1.12**	**1.02-1.24**	**0.015**	**1.12**	**1.02-1.23**	**0.011**
6-10 partners	0.83	0.61-1.13	0.246	0.85	0.62-1.16	0.311
> 10 partners	0.81	0.63-1.04	0.105	0.81	0.63-1.04	0.104
No. of weekly intercourse						
0-1 intercourse	Ref					
2-4 intercourses	NC					
5-10 intercourses	NC					
> 10 intercourses	NC					
> 10 vs. 0-1 (only calculable)	1.03	0.88-1.14	0.961			
No. of deliveries in all partnerships (continuous var)*	1.06	0.98-1.15	0.107			
Practices of oral sex						
No	Ref					
Yes	1.02	0.87-1.20	0.739			
Practices of anal sex						
No	Ref					
Yes	1.22	0.97-1.55	0.087	1.16	0.83-1.62	0.375
Initiation of OC usage						
Above 20 years	Ref					
Below 20 years	**1.32**	**1.04-1.23**	**0.004**	**1.13**	**1.02-1.26**	**0.017**
OC use (Y/N)						
Never use	Ref					
Ever use	1.01	0.74-1.37	0.948			
Smoking habits						
Never smoker	Ref					
Ever smoker	1.06	0.92-1.21	0.384			
Initiation of smoking						
10-13 years of age						
> 13 years of age	**1.16**	**1.01-1.35**	**0.028**	1.11	0.95-1.31	0.159
History of STD						
Yes	Ref					
No	1.06	0.89-1.27	0.505			
History of genital warts						
No	Ref					
Yes	0.93	0.81-1.08	0.372			
History of oral warts						
Yes	Ref					
No	1.08	0.79-1.32	0.823			
2nd pregnancy during FU						
No 2^nd ^pregnancy during FU	Ref					
Yes, 2^nd ^pregnancy during FU	**0.29**	**0.16-0.55**	**0.0001**	**0.32**	**0.17-0.61**	**0.0001**
Change in marital status during FU*						
No	Ref					
Yes; living with partner	0.74	0.41-1.32	0.315	0.78	0.48-1.28	0.340
Yes, married	1.04	0.89-1.22	0.572	1.10	0.84-1.45	0.474
Yes, divorced	**1.11**	**1.01-1.22**	**0.019**	1.16	0.97-1.40	0.102
No. of current sexual partners*						
No	Ref					
1 or more	**1.02**	**1.00-1.04**	**0.027**	**		

Species 7 HPV genotypes: 18,39,45,59,68,70,85; Species 9 HPV genotypes: 16,31,33,35,52,58,67; ^a^Count outcome (incident events/person time at risk), as defined by the first incident event in baseline HPV-negative women during the follow-up; ^b^Results obtained from panel Poisson regression for count outcomes (log-link), clustered by woman-ID, FU visits as time variable, and 95%CI calculated by robust variance estimation; @adjusted for age and all significant univariates in the model; *the variables that are from the FU questionnaire; NC; no convergence obtained (with any working correlation structure) WNL, within normal limits; **rejected because of collinearity

### Statistical analyses

All statistical analyses were run using SPSS^® ^(SPSS, Inc., Chicago, USA) and STATA (Stata Corp., College Station, TX, USA) software packages (PASW Statistics for Windows, version 18.0.1 and STATA/SE 11.0). Frequency tables were analyzed using the χ^2^-test, with the likelihood ratio or Fisher's exact test for categorical variables. Differences in the means of continuous variables were analyzed using non-parametric (Mann-Whitney or Kruskal-Wallis) tests for two- and multiple independent samples, respectively.

### Outcomes of HPV-infection and type-specific incident infection

**Figure 2:**
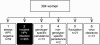
Outcome of the HPV infections during follow-up*. *The genotype-specific outcome of HPV infection in each woman was assessed by comparing the viral events at each FU visit to the baseline HPV status, and 6 different main outcomes were identified. This study evaluated the incidence of genotype-specific HPV infection, women in the second outcome.

The genotype-specific outcomes in each woman were assessed by comparing the viral events at each FU visit to the baseline HPV status and are presented in Figure 2. The following 6 different outcome patterns were defined: 1)always negative, 2)incident HPV, 3)type-specific persistence, 4)non type-specific persistence, 5)fluctuation, and 6)clearance (= transient). The present study focused on those 203 baseline-negative women who developed an incident HPV-infection during the FU (outcome 2) (= women who were HPV-negative at baseline and acquired incident HPV infection during the FU). Genotype-specific persistence denoted for any case with two (or more) consecutive FU-samples positive for the same individual genotype as a single infection or as a part of multiple-type infection. Non-genotype specific persistence includes all cases with two (or more) consecutive samples positive for different HPV genotypes. Clearance was defined as an event (at any FU visit) when a previously HPV-positive test turned out to be negative and remained HPV-negative until the end the FU. Fluctuation is a pattern where consecutive samples are intermittently HPV+ and HPV- with different HPV genotypes, without any two consecutive samples positive for the same or different viral genotype.

Times in months to incident infections were calculated from baseline visit to the first incident event separately for actuarial and crude times. To calculate the former, all baseline HPV-negative women (n = 255) were included in Kaplan-Meier analysis, cases being censored at the first incident event (those who had) or at the end of FU (those who did not develop incident event). Only those women who had completed at least two FU visits were included to this study. To calculate the crude incidence times, only those women with an incident infection with an individual genotype (or species) were included. Both actuarial and crude incidence rates (IR) were calculated, and expressed per 1000 women months at risk (wmr). To get genotype-specific actuarial IRs, the number of incident events for each individual genotypes (and species) was divided by the total wmr (6,212 months for all baseline HPV-negative women), including also the women with no incident event. To calculate crude IRs, only the women with incident events were included and the number of incident events for each individual genotype (or species) was divided by the wmr of those women only. To compare the individual IRs, the rate ratio statistics (STATA) was used, with test-specific 95% confidence intervals (95%CI).

### Predictors of type-specific incident HPV-infections

To analyze the predictors of incident HPV-infections, we used the species-specific incident infection approach to avoid stratifying the material into individual genotypes with single or few cases only. Furthermore, we were only interested in incident HPV-infections by the key HR-HPV-types, i.e., those of Species 7 and Species 9. Women that had an incident infection recorded by a type or types from species 7 and/or 9 were included to these analyses (n = 133).

Poisson regression analysis was used for panel data, clustered by mother-ID and executed (in univariate and multivariate-mode) using population-averaged (PA) model [26,27]. FU visit was the time variable in the panel settings, and incident HPV-infection (count variable) as the dependent variable, with Poisson log-link function. The independent within-group correlation structure for PA model, with robust variance estimator (of 95%CI) to account for within-subject correlation was the best-fitted covariance pattern, defined by QIC (quasi-likelihood information criterion [27]. With these options, Poisson regression for panel data is similar to PA GEE-model, both giving identical results.

In univariate Poisson, we first tested all covariates recorded at baseline questionnaire as well as some selected variables from the FU questionnaire (e.g. a new partner), previously implicated as potential risk factors of HPV in this cohort [18,28]. In the final multivariate model, only variables (as entities, not as sub-categories) that were significant (or borderline significant) in univariate Poisson were entered and were adjusted for age at study entry. All statistical tests were two-sided and declared significant at p-value < 0.05 level.

## Results

### Type-specific incident infections

Of the 255 baseline HPV-negative mothers enrolled in the cohort, 203 experienced an incident event during the FU. The mean follow-up time of these 203 mothers was 58.6 ± 25.2 (SD) months (median 64.3; range 6-94.5). HPV16 caused most frequently incident infection (47.8%, n = 97), followed by HPV18 (3.9%, n = 8), HPV70 (3.0%, n = 6), HPV6 and HPV45 (both 2.5%, n = 5). Multiple-type infections were the second most common incident events after HPV16, with 25.1% (n = 51) frequency. HPV16 and HPV18 were detected in 70.6% (n = 36) and 27.2% (n = 14) of the multiple-type infections, respectively.

The mean times to the first incident event are shown in Table 2. The longest actuarial mean time (75 months) was associated with a single case of HPV33. Of the types with more than one case, HPV31 and HPV45 had actuarial mean times of 34.5 months and 32.8 months, respectively, while the actuarial mean times of HPV-types 6,16,18,35,56,58 varied between 21.5-29 months.

**Table 2:** Times to incident infections and incidence rates for different HPV genotypes and species^a^

	Incident Infections		Mean Time to 1^st ^Incident Infection (months)		Incident Rate (IR) per 1,000 women months at risk (wmr)	
**HPV Genotype:**	**N**	**%**	**Actuarial**^**b **^**(95%CI)**	**Crude**^**c **^**(95%CI)**	**Actuarial**^**b **^**(95%CI)**	**Crude**^**c **^**(95%CI)**

HPV6	5	2.5	27.2 (9.2-45.2)	15.2(10.1-20.2)	0.8 (0.09-1.51)	65.9 (10.0-121.5)
HPV11	2	1.0	12.8 (12.2-13.4)	12.8(12.2-13.4)	0.3 (0.01-0.76)	78.2 (2.5-179.3)
HPV16	97	47.8	25.2 (22.0-28.3)	23.1(20.2-26.0)	15.6 (12.5-18.6)	43.3 (34.8-51.7)
HPV18	8	3.9	27.1 (13.8-40.3)	20.5(14.2-26.8)	1.3 (0.39-2.17)	48.7 (15.8-81.7)
HPV31	4	2.0	34.5 (7.4-61.6)	20.1(6.1-34.1)	0.6 (0.01-1.27)	49.8 (22.4-97.7)
HPV33	1	0.5	75.0 (75.0-75.0)	75.0(75.0-75.0)	0.2 (0.01-0.47)	13.3 (1.3-39.3)
HPV35	2	1.0	21.5 (7.7-35.3)	12.9(11.7-14.1)	0.3 (0.01-0.76)	76.9 (25.5-179.3)
HPV44	1	0.5	12.5 (12.5-12.5)	12.5(12.5-12.5)	0.2 (0.01-0.47)	80.0 (6.8-221.8)
HPV45	5	2.5	32.8 (13.9-51.6)	13.7(6.7-20.6)	0.8 (0.09-1.51)	72.4 (11.2-133.6)
HPV51	2	1.0	13.4 (0-35.7)	13.4(0-35.7)	0.3 (0.01-0.76)	74.1 (24.7-172.8)
HPV52	3	1.5	9.4 (2.0-16.8)	9.4(2.0-16.8)	0.5 (0.06-1.02)	107.1(7.4-221.7)
HPV56	2	1.0	25.4 (7.7-43.1)	42.4(0-101.0)	0.3 (0.01-0.76)	23.5 (8.6-55.7)
HPV58	4	2.0	29.0 (1.0-57.0)	13.5(4.1-22.8)	0.6 (0.01-1.27)	74.1 (42.2-143.9)
HPV59	3	1.5	17.0 (2.0-32.1)	17.0(1.9-32.1)	0.5 (0.06-1.02)	58.8 (5.8-123.4)
HPV66	2	1.0	7.6 (0-17.8)	7.6(0-17.8)	0.3 (0.01-0.76)	133.1 (38.2-305.0)
HPV70	6	3.0	11.2 (2.9-19.4)	6.3(2.0-10.6)	1.0 (0.19-1.73)	157.8 (41.9-273.8)
HPV73	2	1.0	7.6 (0-18.4)	7.6(0-18.4)	0.3 (0.01-0.76)	133.3 (38.7-305.3)
HPV82	3	1.5	9.4 (2.3-16.6)	9.4(2.3-16.6)	0.5 (0.06-1.02)	107.1 (74.1-221.7)
Multiple types	51	25.1	32.1 (26.2-37.9)	19.8(16.4-23.2)	8.2 (5.96-10.45)	50.5 (36.9-63.9)

**HPV Species:**						

Species 5 (26,51,69,82)	5	2.5	11.0 (2.7-19.3)	11.0(2.7-19.3)	0.8 (0.09-1.51)	90.9 (14.9-166.9)
Species 6 (30,53,56,66)	4	2.0	16.5 (3.5-29.4)	25.0(0-56.3)	0.6 (0.01-1.27)	40.0 (1.6-78.4)
Species 7 (18,39,45,59,68,70,85)	22	10.8	27.5 (17.3-37.7)	14.6(10.5-18.7)	3.5 (2.1-5.0)	68.5 (40.9-96.2)
Species 9 (16,31,33,35,52,58,67)	111	54.7	27.5 (23.3-31.7)	22.6(19.7-25.4)	17.9 (14.6-21.2)	44.3 (36.3-52.4)
Species 10 (6,11,13,44,55,74)	8	3.9	27.0 (11.0-42.9)	14.2(11.2-17.4)	1.3 (0.39-2.17)	70.2 (23.3-117.0)
Species 11 (34,73)	2	1.0	7.6 (0-18.4)	7.6(0-18.4)	0.3 (0.01-0.76)	133.3 (38.7-305.3)

^a ^among the 203 women of the Finnish Family HPV study who had an incident infection during the FU; ^b^all baseline HPV-negative women at risk, months at risk calculated 1) until the first incident event, or 2) as total FU months for those with no incident event (Total wmr = 6,212); ^c^only women with an incident event (months at risk calculated until the first incident event);

Apart from the single case of HPV33, HPV56 showed the second longest crude mean time of 42.4 months, followed by HPV16, 18 and 31, with 23.1, 20.6 and 20.1 months, respectively. Remaining genotypes had crude mean times between 6.3 and 17 months.

Species 9 was the most dominant species covering 54.7% (n = 111) of all incident infections and had actuarial mean time of 27.5 months (95%CI23.3-31.7) and crude mean time of 22.6 months (95%CI19.7-25.4). The second and third most common species were species 7 and species 10, with 10.8% (n = 22) and 3.9% (n = 8) prevalence, respectively. Actuarial mean time for species 7, 9 and 10 was similar, 27.5, 27.5 and 27.0 months, respectively. Species 6 and 9 had the longest crude mean incidence times, 25.0 and 22.6 months, respectively.

**Figure 3:**
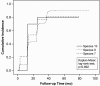
**Cumulative incidence of species 7, 9 and 10 HPV infections in univariate survival (Kaplan-Meier) analysis**.

The cumulative incidence of species 7 and 9 was compared with species 10 in univariate survival (Kaplan-Meier) analysis (Figure 3). Despite a lower cumulative incidence rate for species 10 (benign types), the difference was not statistically significant when compared to species 7 and 9 (log-rank test; p = 0.892).

### Incidence rates

Of single genotypes, HPV16 had by far the highest actuarial IR, 15.6/1000 wmr (95%CI12.5-18.6), followed by multiple-type infections, with 8.2/1000 wmr (95%CI5.96-10.45). Actuarial IR for HPV18 was markedly lower, 1.3/1000 wmr (95%CI0.39-2.17). Due to the dominant role of HPV16, species 9 showed the highest IR of 17.9/1000 wmr (95%CI14.6-21.2), far exceeding that (3.4/1000 wmr) of species 7 (Table 2).

The highest crude IR was ascribed to HPV70, accumulating incident events at a rate of 157.8/1000 wmr. This was followed by HPV73, HPV66, HPV82 and HPV52, with IRs of 133.3/1000 wmr, 133.1/1000 wmr, 107.1/1000 wmr and 107.1/1000 wmr, respectively. The crude IRs of HPV16 and HPV18 were far lower, and practically identical to each other, 43.3/1000 wmr and 43.4/1000 wmr, respectively. Due to this wide variation among individual types included in different species, the crude IRs between the HPV-species showed much less variation, i.e., from 40/1000 wmr to 91/1000 wmr, when species 11 (HPV73 alone) is excluded (Table 2).

### Predictors of species-specific incident infections

The predictors of genotype-specific incident infections of species 7 and 9 are shown in Table 1. In univariate Poisson regression, seven variables in total were significantly associated with incident infection. The following four variables were associated with increased risk: 1) mother being seroconverted to HR-HPV during FU, 2) < 13 years of age at onset of sexual activity, 3) 1-2 lifetime sexual partners 4) ≥2 sexual partners during FU. The other three variables were associated with a decreased risk of incident infection: 5) later initiation of oral contraceptives (OC)(age > 20 years), 6) age at initiation of smoking (age10-13 years) and 6) pregnancy at FU-visit.

When all significant and borderline significant variables were entered in the multivariate Poisson PA model, together with mother's age, three variables retained their significance as independent factors with lower risk for incident species 7 and 9 infection during the FU: 1) > 2 life-time sexual partners (p = 0.011), 2) late (> 20 years) initiation of OCs (p = 0.017), and 3) pregnancy at FU-visit (p = 0,0001), with the details of estimates in sub-categories shown in Table 1.

## Discussion

This is one of the first studies to assess type-specific incident HPV-infection [8-16], in contrast to the previous studies, where acquisition of HPV-infection has been analysed collectively for all HPV-types or acquisition of HR- and LR-types has been compared [4-7]. Similarly, in currently available literature, it is hard to find the times to the first incidence infections at the genotype-level, most reports being focused on duration of incident infections [8,9,16].

We previously analysed crude incidence times collectively for 13 HR-HPV-types detected by Hybrid capture 2-assay [6]. It took an average of 16.6 months to develop the first incident HR-HPV event. In the present study, we went further and provide these times for individual HPV-genotypes (and species) both as actuarial and crude format. To calculate the former, included are also baseline HPV-negative women who never develop an incident event, while the latter is calculated only for women who develop incident events. Thus, the actuarial times reflect the real-life situation, while indicating how long it takes in a cohort of baseline HPV-negative women to develop the first incident infections. On the other hand, the crude times indicate the time that these first incident infections take to develop among women who all experience an incident event. These crude (type-specific) incidence times provide a robust measure to compare different HPV-genotypes in their speed to develop the first incident events (Table 2). In this respect, individual genotypes showed marked differences in their crude incidence times, varying within the range of 2 to 90 months, with the mean of 20.3 and median of 13.5 months (data not shown). Generally speaking, LR-HPV-types had shorter crude mean incidence times than HR-HPV-genotypes. However, despite the significant overall difference (p = 0.0001) between the individual genotypes, these results are hampered by multiple comparisons and a small number of cases in each strata. Of interest is the observation that no significant differences were observed between mean incidence times of HPV16, HPV18 and multiple-type infections, each being represented by sufficient number of cases.

As evident in Figure 3, these incident events show a tendency for clustering around the time points of the control visits, i.e., at 2-, 12-, 24-, 36- months and 6 years. This flaw is inherent to the study design, however, and equally affects all studies with similar design, i.e., HPV sampling at irregular (and relatively long) intervals. Because of the fact that the incident events rarely (if ever) occur exactly at the time point of sampling, but somewhere in between the two subsequent samplings, and because the event leads to censoring of the subject (= no longer at risk for incident event), we can speak about interval censoring. Due to the fact that there are both "left-censored and right-censored" (relative to the point) data that occur at random, we feel that this should not cause a major bias, however. Of course, the exact times can only be obtained by very short (1 to 3 months) sampling times, which in the case of HPV (having protracted clinical course) are not feasible in practice.

Also the IR can be calculated as actuarial and crude, as done here. These two IRs are markedly different, because of the significantly different person months at risk. This also makes comparison between individual studies difficult, particularly when the FU-times and cohort size are substantially different. If one compares a setting where 1000 women are followed-up for 10 months (10.000 wmr), and another one with 100 women followed-up for 100 months (10.000 wmr), certainly the IRs in the latter are higher. This is because the median incidence time is around 12-13 months and only a fraction of events can be detected with a short FU-time. In addition, it is essential to know the age-profile of the cohort, because IR for HPV infection is critically age-dependent [7].

In our cohort, actuarial IR was by far the highest for HPV16, 15.6/1000 wmr. This is consistent with other studies, where HPV16 had the highest actuarial IR, albeit the absolute IRs of these cohorts was much lower. A young women's health study-cohort from USA reported an actuarial IR of 5.9/1000 wmr for HPV16 [9]. In the Ludwig-McGill low-income cohort, the actuarial IR for HPV16 was 1.4/1000 wmr [8], which is very similar to another cohort consisting of sexually active women from Hawaii, 1.77/1000 wmr [16]. Due to the dominant role of HPV16, also the actuarial IR of species 9 was the highest 17.9/1000 wmr, which is three times higher than recently published by Goodman and co-workers. (5.4/1000 wmr) [16]. As pointed out above, these differences are explained by different cohort sizes and particularly by much shorter FU-times of the other studies, 10-15 months only [8,9,16]. Another factor is the different age-profile; the mean age of our cohort was 25.5 years which is close to 24.2 years in the study by Giuliano and co-workers [9] where as the mean age in the study by Goodman and co-workers was 35 years [16]. The IRs are higher in our cohort and Giuliano cohort [9] than in studies on older women [8,16], which gives further support to close age-dependence [6,7].

This is one of the first studies on crude IRs, which reflect the differences in the rates at which individual genotypes accumulate incident events among women who have these events. Although firm conclusions on single genotypes are difficult because of the rarity of many individual genotypes, it is interesting to note that the crude IR of the key LR-HPV-types (HPV6,11), is almost twice as high as that of the two main HR-types (HPV16,18). This indicates that the former develop incident infections at much higher rate, which is also shown by their significantly shorter times to incident events (Table 2). Using rate-ratio (RR) statistics, one cannot detect significant differences between the different HPV-species in their crude IRs (data not shown). Much larger cohort is needed to establish whether such differences exist between individual HR-HPV-genotypes. If confirmed, however, such data would have important implications e.g. in the selection of the most aggressive genotypes with high IRs as targets of the primary prevention.

As to the risk-factors for incident species 7/9 infections, seven variables were significant predictors in univariate analysis. First of these was linked with HPV-serology [25]; mothers who failed to seroconvert to HR-HPV during the FU were at increased risk for incident infections (Table 1). It is well established that serological response to HPV-infections needs several months to become detectable [25,29]. Thus, women who experience an incident event close to the end of FU have not enough time to become seroconverted during the observation period. There is some evidence that seroconversion may fail with transient and even with some persistent infections [30]. It is commonly believed that high levels of HPV-antibodies detected after seroconversion will protect against a new infection. Thus, seroconversion detected during the early months of FU could implicate an incident event that took place before the baseline visit, and these high antibody levels, except for being incompatible with new incident events, might also neutralise the virus and make these women baseline HPV DNA-negative.

Initiation of smoking beyond 13 years of age was associated with an increased risk of incident HPV-infection, but lost its significance in multivariate model. Some studies have found no association between smoking and incident infection [10,16], whereas others indicating current smoking to increase the risk [5,11,13,15]. Our recent analysis disclosed that initiation of smoking at < 13 years of age increased the risk of type-specific persistence [31]. Because incident infections precede viral persistence, the present data are consistent with this observation, implicating that women who start smoking later have had less time to develop persistent infection, and consequently, are at the phase when incident infections accumulate.

Women who started their sexual activity before 13 years of age were found to have a lower risk for incident infections during the FU. Also this association is in line with our recent analysis disclosing early age of sexual debut as a risk factor for HR-HPV persistence [31]. It is likely that women with later onset are at increased risk for incident infections, just because those with longer sexual exposure have already experienced infection, and either cleared or developed a persistent infection by the time they reach their 20s (mean age of this study cohort being 25.5 years).

The same analogy applies to life-time number of sexual partners (also a proxy to HPV exposure), shown in this study to be inversely related to the risk of incident infections, i.e., lower partner number was associated with an increased risk. It seems likely that those with more partners have already experienced such an event before enrolled in the study, i.e., are baseline HPV+ and by definition, no longer at risk for incident infection by the same HPV genotype.

The initiation of OC usage before the age of 20 increased the risk for incident infection and was a significant predictor also in the multivariate model. Previous studies report conflicting results; current use of OCs has been shown to increase [11] or decrease [4] the risk of incident infection. In addition, it has been reported that the current users of OCs were not in increased risk and that the past users of OCs seemed to be protected, as shown by a decrease of incident infections with the years of OC use [16]. Contradictory to the previous finding, a recent study stated that the risk for incident infection increased with increasing years of OC use [13]. It is obvious that further studies are needed to evaluate the association of OC with incident infections.

Another significant protective predictor was the second pregnancy during the FU (Table 1). No previous data are available from a similar setting where newly delivered mothers were prospectively followed-up. We have recently shown that women committed to the second child did not share many of the known life-style behavioural risk factors of HPV-infection [32], and this could be the likely explanation for this significant (IRR = 0.32,95%CI0.17-0.61) protective effect of a new pregnancy against incident species 7/9 HPV-infections in the present analysis. Some recent data suggest that parity was protective especially against LR-HPV-types [10]. In our cohort, increasing parity did not show any such effect. However, in a large screening study (the NIS Cohort), ever being pregnant was an independent predictor of incident infection [6].

The main limitations of this current study are firstly the small cohort size of the women; the total number of incidence infections was 203. To gain more information on genotype specific incident HPV-infections, times and rates, and to increase the statistical power, a much larger cohort is needed. Secondly the women were all pregnant at the time of enrolling to this study, as it is the special strength of this study it also may be considered as a limitation because the detailed mechanism how pregnancy and HPV infections interacts are not that well understood.

## Conclusions

Data obtained from this study indicate that the probability of mothers, who test HPV-negative just before delivery, to contract an incident infection by species 7/9-genotypes during the subsequent follow-up is less likely among those who report more than 2 life-time sexual partners at baseline, start using of OCs after age 20, and become pregnant for the second time during the follow-up. To the best of our knowledge, this is the first study where actuarial and crude times to the incident events as well as actuarial and crude IRs are evaluated at the genotype- and HPV-species level. Obtaining genotype-specific data during long-term follow-up is needed for better understanding of the natural history of genotype specific HPV-infections, e.g. the times and rates at which each genotype accumulates incident events. It sounds feasible to reason that in the future, those genotypes with shortest times to incident infections (i.e., the highest IRs), could be the most appropriate targets for early prevention, e.g. by genotype-specific multivalent HPV vaccines.

## Competing interests

The authors declare that they have no competing interests.

## Authors' contributions

KL is the first author in this study, participated in execution of the work at all phases, this study is part of her PhD project. KS is a pathologist and senior author in the project, with main responsibility in maintaining the databank as well as designing and running the statistical analyses. SG is the clinical principal investigator and coordinator at the Department of Obstetrics and Gynecology, and has participated in the design of the study and analysis and writing of the results. MR has been responsible for enrolment and examination of all mothers during the follow-up and writing of the results. SS is the coordinator and principal investigator of the Finnish Family HPV study and the head of the HPV research laboratory. All authors have read and approved the final version of this manuscript.

## Pre-publication history

The pre-publication history for this paper can be accessed here:

http://www.biomedcentral.com/1471-2334/11/179/prepub
